# pfsearchV3: a code acceleration and heuristic to search PROSITE profiles

**DOI:** 10.1093/bioinformatics/btt129

**Published:** 2013-03-16

**Authors:** Thierry Schuepbach, Marco Pagni, Alan Bridge, Lydie Bougueleret, Ioannis Xenarios, Lorenzo Cerutti

**Affiliations:** ^1^Vital-IT Group, SIB Swiss Institute of Bioinformatics, Genopode, UNIL-Sorge, 1015 Lausanne and ^2^Swiss-Prot Group, SIB Swiss Institute of Bioinformatics, CMU, 1 rue Michel-Servet, CH-1211 Geneva 4, Switzerland

## Abstract

**Summary:** The PROSITE resource provides a rich and well annotated source of signatures in the form of generalized profiles that allow protein domain detection and functional annotation. One of the major limiting factors in the application of PROSITE in genome and metagenome annotation pipelines is the time required to search protein sequence databases for putative matches. We describe an improved and optimized implementation of the PROSITE search tool pfsearch that, combined with a newly developed heuristic, addresses this limitation. On a modern x86_64 hyper-threaded quad-core desktop computer, the new pfsearchV3 is two orders of magnitude faster than the original algorithm.

**Availability and implementation:** Source code and binaries of pfsearchV3 are freely available for download at http://web.expasy.org/pftools/#pfsearchV3, implemented in C and supported on Linux. PROSITE generalized profiles including the heuristic cut-off scores are available at the same address.

**Contact:** pftools@isb-sib.ch

## 1 INTRODUCTION

Falling costs and continuing technological developments have led to a dramatic increase in the rate of sequencing of individual species genomes (Lindblad-Toh *et al.*, 2011) and the diversity of the ecological niches sampled by metagenomic sequencing (Teeling and Glöckner, 2012). The identification, classification and functional annotation of the putative protein sequences encoded by these samples is essential to understand the diversity of the underlying biological systems, and will ultimately allow the construction of biological models that simulate and make testable predictions about their behaviour (Faust and Raes, 2012).

Most functional annotation is predicted using sequence homology-based methods that infer the function of uncharacterized protein sequences based on their similarity to characterized templates. These methods include generalized profiles and Hidden Markov Models (HMMs), which can detect more subtle homologies than pairwise sequence alignments (Park *et al.*, 1998). The application of these computationally expensive methods on large datasets has been made feasible by the development of heuristics for sequence database search and faster more efficient code (e.g. Eddy, 2011).

Our PROSITE method combines manually constructed generalized profiles for efficient domain detection with rules for precise functional annotation (Sigrist *et al.*, 2013). Here, we describe a new heuristic method and code optimization and parallelization for the PROSITE profile-sequence database search tool pfsearch. These developments increase the speed of pfsearch by two orders of magnitude using a modern x86_64 hyper-threaded quad-core computer (see Table 1 legend for specifications of the computer used in our tests), making the annotation of large sequence datasets using PROSITE feasible.

**Table 1.** Execution times to search the PROSITE profile PS50255 (CYTOCHROME_B5_2) against 16 544 936 UniProtKB sequences (5 358 014 649 residues)

	−*heuristic*		+*heuristic*	
	SSE2	SSE4.1	SSE2	SSE4.1
pfsearch (v2.4)	51m32s	n.a.	n.a.	n.a.
pfsearchV3 (1 core*)	33m02s	20m17s	1m55s	1m44s
pfsearchV3 (2 cores*)	16m54s	10m23s	0m58s	0m53s
pfsearchV3 (4 cores*)	9m14s	5m40s	0m31s	0m28s
pfsearchV3 (8 cores^+^)	9m04s	5m28s	0m28s	0m27s

The pfsearch and pfsearchV3 programs have been compiled on a Gentoo Linux (-mtune = corei7 -march = corei7 -fomit-frame-pointer -O2) with gcc (4.6.3) and glibc (2.15) using the following compilation options: -O3 –enable-mmap –enable-thread-affinity, CFLAGS = ‘-mtune = corei7 -march = corei7 -ffast-math -mfpmath = sse’, FFLAGS = ‘-mtune = corei7 -march = corei7 -ffast-math -mfpmath = sse’. The static executable is available at the provided WEB address. All run times have been measured on a quad-core Intel® Core^TM^ i7-3770 CPU @ 3.40 GHz with 8 Gb RAM running on Linux 3.2.0-4-amd64. The number of cores, the selection of the SSE and the selection or otherwise of the heuristic where specified at runtime with options -t, -s and -C, respectively, of pfsearchV3. Both pfsearch and pfsearchV3 have been run to produce the same output alignment, options -fxzl and −o 2 respectively. (*) physical cores obtained with option -k and -t of pfsearchV3. (+) the default mode of pfsearchV3, which uses all available cores with hyper-threading for a total of eight cores in our testing machine (no options -t and -k are used). NB: pfsearchV3 was run using an indexed sequence database (option -i); selecting this option reduces the execution time by 7 s in all experiments using the specified set of protein sequences.

## 2 RESULTS AND DISCUSSION

### 2.1 Heuristics for generalized profiles

A major reduction in the execution time of sequence database searches can be achieved by an heuristic filter that selects sequences for the next CPU-expensive alignment step of the core algorithm. One such heuristic is the MSV algorithm of HMMER3, which computes the sum of multiple optimal un-gapped local alignment segments (Eddy, 2011). Although extremely fast and convenient, the MSV heuristic filter cannot be directly transposed to generalized profiles that have a different model topology and are not bound to the probabilistic model restrictions of HMMs. We therefore developed a variant that is directly applicable to generalized profiles.

Our pfsearch heuristic, named *prfh*, sums the maximal matching diagonals between the profile and the sequence, ignoring both gaps and the order of the matching diagonals. First, for each position *i* of the profile and *j* of the sequence, we define a score *S*(*i,j*):
(1)
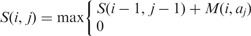

where *M*(*i,a_j_*) is the match score read at position *i* of the profile matrix table for residue *a_j_* observed at position *j* of the sequence. Boundary scores *S*(*i*,0) and *S*(0,*j*) are set to 0. Second, only the maximal scoring diagonal *S*(*i*,*j*) is kept for every position *j* of the sequence [the maximization part of Equation (2)]. All maxima are then summed to form the final heuristic score (*H_score_*).
(2)
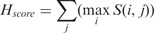



**Fig. 1.**
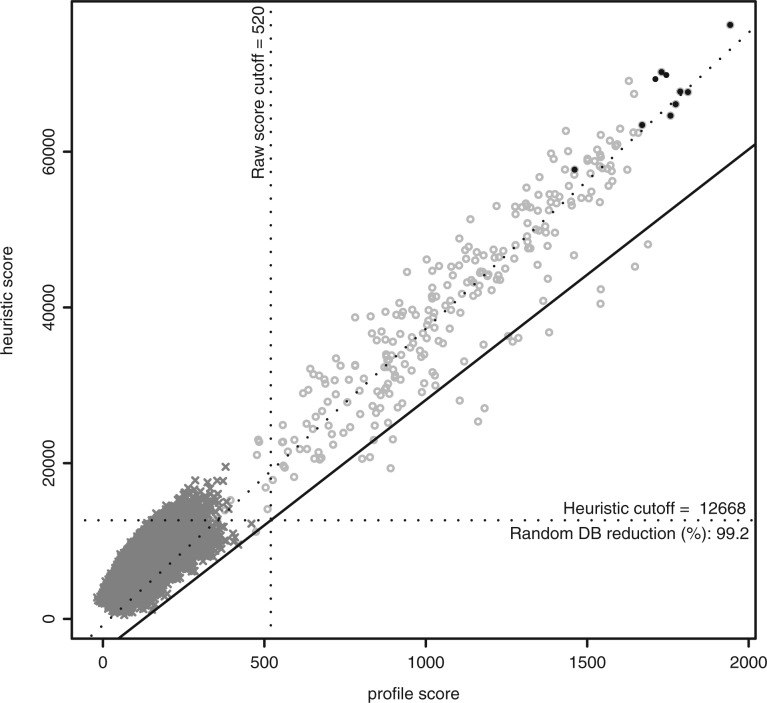
Estimation of the heuristic score cut-off for the PROSITE profile PS50255 (CYTOCHROME_B5_2). The profile scores and heuristic scores are plotted for the matched sequence: (closed circle) sequences from the seed alignment; (multi symbol) shuffled UniProtKB/Swiss-Prot sequences; (open circle) simulated sequences derived from the seed alignment mutated at various PAM distances (see text for explanatory notes). The heuristic search scores and profile search scores of the simulated sequences (open circle) exhibit a strong positive correlation (*R*^2 ^= 0.9). These scores are used to estimate the linear regression for the lower 5% quantile (black line) used to map the profile search scores to heuristic search scores. The standard linear regression is also plotted (dashed line)

The *H_score_* distribution measured using PROSITE profiles on UniProtKB linearly correlates with the raw score distribution obtained using the standard pfsearch (*R*^2^ ≈ 0.9 on average). To determine the appropriate *H_score_* cut-offs with respect to the normalized score cut-offs of each calibrated profile (Sigrist *et al.*, 2002), we apply the following procedure. We randomly sample 200 sequences belonging to the original seed alignment for each profile (re-sampling if their number is <200), and generate a set of artificially mutated sequences from these, including indels, at various PAM distances. These artificial sequences (sharing from 40–85% sequence identity with their source) are then scored with both the standard profile scoring method and the heuristic (Fig. 1). We calculate the regression line on the lower 5% quantile of the heuristic score distribution using the quanteg R package (http://cran.r-project.org/web/packages/quantreg/), and use it to obtain the heuristic cut-offs corresponding to the standard profile cut-offs (Fig. 1). The regression on a low quantile ensures a minimal loss of true-positive sequences.

This method to fix the *H_score_* cut-offs was automatically applied on the PROSITE profiles. Manual inspection showed that this method was appropriate for the majority of the profiles, although in some cases, the *H_score_* cut-off could be manually increased to further accelerate the search. A minority of very short or ‘exotic’ profiles cannot be used with the heuristic. For these, no *H_score_* cut-off is defined in the profile, and the pfsearch software skips the heuristic search step.

The heuristic reduces the mean search database size by 96.7% (median 99.1%). The recovery of true positives is ≥98% for >99% of the PROSITE profiles with an associated *H_score_* cut-off (the lowest measured recovery is 92.6%). The majority of the missing true positives correspond to fragmentary sequences in UniProtKB.

### 2.2 Software optimization and performance of the new pfsearch

Pfsearch has been rewritten and optimized in C from the original Fortran. The code will run on any x86_64 POSIX compliant architecture and benefits from the SSE 4.1 instruction set when available. However with the current source code, only Linux operating systems may benefit from CPU core affinity and file to memory mapping optimization, detected at compile time. The optimization process entirely reformatted the memory structure to allow vectorization. High level assembly code (intrinsic functions) was used to enforce the SSE2 and SSE4.1 instruction sets, leading to a 2-fold acceleration of the original Fortran (Table 1). SSE4.1 is particularly effective in reducing the execution time of the core pfsearch algorithm, while both SSE4.1 and SSE2 show similar performance on the heuristic filter (Table 1). This acceleration scales up with multithreading: on a dual hyper-threaded quad-core machine, we measured an average 10-fold improvement. The scaling is clearly related to the number of physical cores, with hyper-threading having only a minimal effect on performance (Table 1).

Multithreading implementation is straightforward because profile alignment versus a database is in itself an embarrassingly parallel task. For pfsearchV3, we implemented a master–slave mechanism to analyse and adapt the load before each phase of the algorithm (heuristic, filter, alignment), thus providing more equitable shares between threads. This has some constraints: sequences are read several times, but above all, they are no longer accessed sequentially, so an index of the sequences has to be either computed or loaded at start.

By combining the heuristic with our code optimization, we achieved a 100× increase in the speed of pfsearch on average. To search 16 544 936 UniProtKB sequences (5 358 014 649 residues) required a mean of 98 s/profile (median of 73 s/profile). A typical example of the runtime acceleration achieved is shown in Table 1.

The heuristic version of pfsearch can be used to annotate large sets of complete sequences in a reasonable amount of time on a modern workstation. For example, the human proteome can be searched with the totality of the PROSITE profile models in <4 hours, and this time can be drastically reduced on machines with a large number of CPU cores and/or computer clusters. For fragmented sequences, users may inactivate the heuristic to minimize loss of true-positive matches, in which case the speed of execution will be determined by the number of available CPU cores. We also plan to implement our heuristic search method in the HAMAP pipeline that provides high quality functional annotation for protein families (Pedruzzi *et al.*, 2013).
